# Dentin hypersensitivity: Recent trends in management

**DOI:** 10.4103/0972-0707.73385

**Published:** 2010

**Authors:** Sanjay Miglani, Vivek Aggarwal, Bhoomika Ahuja

**Affiliations:** Department of Conservative Dentistry and Endodontics, Faculty of Dentistry, Jamia Millia Islamia, New Delhi - 110 025, India

**Keywords:** Casein phosphopeptide - amorphous calcium phosphate, dentinal hypersensitivity, desensitizing agents, fluorides

## Abstract

Dentinal hypersensitivity (DH) is a common clinical condition usually associated with exposed dentinal surfaces. It can affect patients of any age group and most commonly affects the canines and premolars of both the arches. This article concisely reviews the patho-physiology, mechanism and clinical management of the DH. Treatment of DH should start with an accurate diagnosis. Differential diagnosis should be made and all other probable causes should be excluded. An often neglected phase of clinical management of DH is the identification and treatment of the causative factors of DH. By removing the etiological factors, the condition can be even prevented from occurring or recurring. There are various treatment modalities available which can be used at home or may be professionally applied. The “at home” desensitizing agents include toothpastes, mouthwashes or chewing gums and they act by either occluding the dentinal tubules or blocking the neural transmission. This article also discusses the recent treatment options like bioglass, Portland cement, lasers and casein phosphopeptide.

## INTRODUCTION

Dentine sensitivity (DS) or dentinal hypersensitivity (DH) is one of the most commonly encountered clinical problems. It is clinically described as an exaggerated response to application of a stimulus to exposed dentine, regardless of its location.[1,2] The terms DS or DH have been used interchangeably to describe the same clinical condition. True hypersensitivity can develop due to pulpal inflammation and can present the clinical features of irreversible pulpitis, i.e., severe and persistent pain, as compared with typical short sharp pain of DH.[3] Majority of literature reviews dealing with this clinical condition have suggested the use of term DS and consider that the sharp pain is actually the normal pulpal response to the exposed dentine.[4,5] But it is well known that all exposed dentine are not sensitive and the term DH has been used over the decades by the clinicians.[6,7] Therefore, both the terminologies can be used to describe the clinical condition. The condition has been defined by an international workshop on DH as follows:[8] “Dentine hypersensitivity is characterized by short, sharp pain arising from exposed dentine in response to stimuli, typically thermal, evaporative, tactile, osmotic or chemical and which cannot be ascribed to any other dental defect or pathology”. Some authors have substituted the word “dentine” and added the site, such as cervical or root, resulting in various other terminologies (e.g., cervical sensitivity/hypersensitivity) to describe the same clinical condition.[9]

## PREVALENCE AND EPIDEMIOLOGY

DH is a painful clinical condition with an incidence ranging from 4 to 74%.[10–14] The variations in the reports may be because of difference in populations and different methods of investigations. The methods employed are usually patient questionnaires or clinical examinations. Interestingly, the incidence of DH is much higher in patient questionnaires studies than in clinical studies which quote an incidence of mere 15%.[11,12]

A slightly higher incidence of DH is reported in females than in males. While DH can affect the patient of any age, most affected patients are in the age group of 20–50 years, with a peak between 30 and 40 years of age.[11] Regarding the type of teeth involved, canines and premolars of both the arches are the most affected teeth. Buccal aspect of cervical area is the commonly affected site.[15]

## ETIOPATHOGENESIS

### Anatomy of dentine pulp complex

Dentine is covered and protected by hard tissues such as enamel or cementum. Dentin itself is a vital tissue, consisting of dentinal tubules, and is naturally sensitive because of extensions of odontoblasts and formation of dentine–pulp complex.[16] Although dentin and pulp are histologically different, their origin is embryologically from the same precursor, i.e., the ectomesenchyme.[16] Pulp is integrally connected to dentine, i.e., physiologic and/or pathologic reactions in one of the tissues will also affect the other. Dentin consists of small canal like spaces, dentinal tubules. These tubules occupied by odontoblastic processes.[16] The odontoblastic processes may extend through the entire thickness of dentin from pulp to dentino-enamel junction. The odontoblastic processes are actually the extensions of odontoblasts, which are the major cells of pulp–dentin complex.[16] The odontoblastic processes are surrounded by dentinal fluid inside the tubules. The dentinal fluid forms around 22% of total volume of dentin.[16] It is an ultrafiltrate of blood from the pulp via dentinal tubules and forms a communication medium between the pulp (via the odontoblastic layer) and outer regions of the dentin.

### Pathogenesis

It has been stated in the literature that DH develops in two phases: lesion localization and lesion initiation.[17] Lesion localization occurs by loss of protective covering over the dentin, thereby exposing it to external environment. It includes loss of enamel via attrition, abrasion, erosion or abfraction. Another cause for lesion localization is gingival recession which can be due to toothbrush abrasion, pocket reduction surgery, tooth preparation for crown, excessive flossing or secondary to periodontal diseases.[18] As stated earlier, not all exposed dentine is sensitive. For DH to occur, the lesion localization has to be initiated. It occurs after the protective covering of smear layer is removed, leading to exposure and opening of dentinal tubules.

## MECHANISM

Three major mechanisms of dentinal sensitivity have been proposed in the literature:

- Direct innervation theory
- Odontoblast receptor
- Fluid movement/hydrodynamic theory

According to direct innervation theory, nerve endings penetrate dentine and extend to the dentino-enamel junction.[19] Direct mechanical stimulation of these nerves will initiate an action potential. There are many shortcomings of this theory. There is lack of evidence that outer dentin, which is usually the most sensitive part, is innervated. Developmental studies have shown that the plexus of Rashkow and intratubular nerves do not establish themselves until the tooth has erupted; yet, newly erupted tooth is sensitive.[16] Moreover, pain inducers such as bradykinin fail to induce pain when applied to dentine, and bathing dentine with local anesthetic solutions does not prevent pain, which does so when applied to skin.

Odontoblast receptor theory states that odontoblasts acts as receptors by themselves and relay the signal to a nerve terminal.[20] But majority of studies have shown that odontoblasts are matrix forming cells and hence they are not considered to be excitable cells, and no synapses have been demonstrated between odontoblasts and nerve terminals.[21]

Brannstrom (1964) has proposed that dentinal pain is due to hydrodynamic mechanism, i.e., fluid force.[22] Scanning electron microscopic (SEM) analysis of “hypersensitive” dentin shows the presence of widely open dentinal tubules.[6] The presence of wide tubules in hypersensitive dentin is consistent with the hydrodynamic theory. This theory is based on the presence and movement of fluid inside the dentinal tubules. This centrifugal fluid movement, in turn, activates the nerve endings at the end of dentinal tubules or at the pulp–dentine complex.[21] This is similar to the activation of nerve fibers surrounding the hair by touching or applying pressure to the hair. The response of pulpal nerves, mainly Aδ intradentinal afferent fibers, depends upon the pressure applied, i.e., intensity of stimuli.[21] It has been noted that stimuli which tend to move the fluid away from the pulp–dentine complex produce more pain. These stimuli include cooling, drying, evaporation and application of hypertonic chemical substances.[23] Approximately, 75% of patients with DH complain of pain with application of cold stimuli.[23] In spite of the fact that fluid movement inside the dentinal tubules produces pain, it should be noted that not all exposed dentine is sensitive. As stated before, the “hypersensitive” dentin has more widely open tubules and thin/under calcified smear layer as compared with “non-sensitive” dentine. The wider tubules increase the fluid movement and thus the pain response.[6,7]

## CLINICAL MANAGEMENT OF DH

### Diagnosis

As like any other clinical condition, an accurate diagnosis is important before starting the management of DH. DH has features which are similar to other conditions like caries, fractured or chipped enamel/dentine, pain due to reversible pulpitis, and post dental bleaching sensitivity.[15,24] Diagnosis of DH starts with a thorough clinical history and examination. The other causes of dental pain should be excluded before a definite diagnosis of DH is made. Some of these techniques include pain response upon the pressure of tapping teeth (to indicate pulpitis/periodontal involvement), pain on biting a stick (suggests fracture), use of transilluminating light or dyes (to diagnose fractures), and pain associated with recent restorations.[25] A simple clinical method of diagnosing DH includes a jet of air or using an exploratory probe on the exposed dentin, in a mesio-distal direction, examining all the teeth in the area in which the patient complains of pain.[26] The severity or degree of pain can be quantified either according to categorical scale (i.e., slight, moderate or severe pain) or using a visual analogue scale.[17]

### Prevention of DH/removal of etiological factors

**Figure 1:**
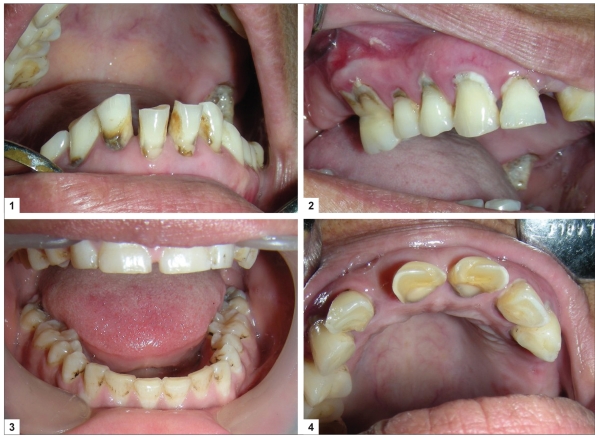
(1) Patient with poor oral hygiene, generalized attrition and deposition of calculus, (2) Excessive scrubbing at cervical areas, leading to abrasion cavities and gingival recession, (3) Generalized attrition, (4) Erosion defects on the palatal surfaces because of endogenous acids

An often, neglected phase of clinical management of DH is the identification and treatment of the causative factors of DH. By removing the etiological factors, the condition can be even prevented from occurring or recurring. The etiological factors include faulty tooth brushing, poor oral hygiene [Figure 1], premature contacts, gingival recession because of periodontal therapy or physiological reasons, and exogenous/endogenous non-bacterial acids.[17]

Faulty tooth brushing includes hard brushes, excessive forces, excessive scrubbing at the cervical areas or even lack of brushing which causes plaque accumulation and gingival recession [Figures 2 and 3].[27] The patient should be taught the correct method of tooth brushing with the help of a model. Highly abrasive tooth powder or pastes should be avoided.[17] Also, the patients should be instructed to avoid brushing for at least 2 hours after acidic drinks to prevent agonist effect of acidic erosion on tooth brush abrasion.

Erosive agents are also important agents in initiation and progression of DH [Figure 4]. They tend to remove the enamel or open up the dentinal tubules.[28,29] The erosive agents can be either exogenous dietary acids or endogenous acids. The exogenous dietary acids include carbonated drinks, citrus fruits, wines, yogurt, and professional hazards (workers in battery manufacturing, wine tasters).[28,29] A detailed dietary history should be taken. The quantity and frequency of the foods containing acids should be reduced. Patient should be advised to take something alkaline (milk) or at least neutral (water) after acidic drinks and to use a straw to sip the drink and avoid swishing it around the teeth. The endogenous acid comes from gastroesophageal reflux or regurgitation. It is also common in patients with eating disorders. The condition is characterized by generalized erosion of the palatal surfaces of maxillary anterior teeth.[30] Such a patient should be referred to the medical practitioner for expert management of the underlying disease. An occlusal splint can be fabricated to cover the affected areas, to prevent their contact with the acids.

## CLASSIFICATION OF DESENSITIZING AGENTS

- **Mode of administration**
  At home desensitizing agents
  In-office treatment
- **On the basis of mechanism of action**
  Nerve desensitization
  - Potassium nitrate
  Protein precipitation
  - Gluteraldehyde
  - Silver nitrate
  - Zinc chloride
  - Strontium chloride hexahydrate
  Plugging dentinal tubules
  - Sodium fluoride
  - Stannous fluoride
  - Strontium chloride
  - Potassium oxalate
  - Calcium phosphate
  - Calcium carbonate
  - Bio active glasses (SiO_2_–P_2_O_5_–CaO–Na_2_O)
  Dentine adhesive sealers
  - Fluoride varnishes
  - Oxalic acid and resin
  - Glass ionomer cements
  - Composites
  - Dentin bonding agents
  Lasers
  - Neodymium:yttrium aluminum garnet (Nd-YAG) laser
  - GaAlAs (galium-aluminium-arsenide laser)
  - Erbium-YAG laser
  Homeopathic medication
  - Propolis

### At home desensitizing therapy

Grossman[31] listed the requirements for an ideal dentine desensitizing agent as: rapidly acting with long-term effects, non-irritant to pulp, painless and easy to apply, and should not stain the tooth. Traditionally, the therapy for management of DH is primarily aimed at occluding the dentinal tubules or making coagulates inside the tubules.[17] Patients are often prescribed over-the-counter desensitizing agents. These “at home” desensitizing agents include toothpastes, mouthwashes and chewing gums.[17] Majority of the toothpastes contain potassium salts (potassium nitrate, potassium chloride or potassium citrate), sodium fluoride, strontium chloride, dibasic sodium citrate, formaldehyde, sodium monofluorphosphate and stannous fluoride. Potassium salts act by diffusion along the dentinal tubules and decreasing the excitability of the intradental nerve fibers by blocking the axonic action.[32,33] Various clinical studies have shown the efficacy of potassium salts in controlling the DH.[34,35] It has been shown that toothpastes containing 5% potassium nitrate and 0.454% stannous significantly reduced the DH. Also, toothpastes containing potassium nitrate and fluorides have been shown to reduce post-bleaching sensitivity.[36,37] The desensitizing toothpastes should be used with the help of a toothbrush with soft bristles. Patients should be advised to use minimal amount of water to prevent the dilution of the active agent. Along with the desensitizing toothpastes, mouthwashes and chewing gums containing potassium nitrate, sodium fluoride or potassium citrate are also recommended.[17] The results of “at-home” desensitizing therapy should be reviewed after every 3–4 weeks. If there is no relief in DH, “in-office” therapy should be initiated.

### In-office desensitizing agents

Theoretically, the in-office desensitizing therapy should provide an immediate relief from the symptoms of DH. The in-office desensitizing agents can be classified as the materials which undergo a setting reaction (glass ionomer cement, composites) and which do not undergo a setting reaction (varnishes, oxalates).

### Fluorides

Traditionally, fluorides have been used as a caries preventive material which can help in remineralization of enamel/dentin.[38] Also, various clinical trials have shown that application of fluoride solution can decrease the DH.[39,40] Fluorides decrease the dentinal permeability by precipitation of calcium fluoride crystals inside the dentinal tubules.[17] These crystals are partially insoluble in saliva. SEM revealed granular precipitates in the peritubular dentin after application of fluorides.[41] Various fluoride formulations are used to treat DH. These include sodium fluoride, stannous fluoride, sodium monofluorophosphate, fluorosilicates and fluoride combined with iontophoresis.[17] Sodium fluoride has been used in dentifrices or may be professional applied in a concentration of 2%. The precipitates formed by sodium fluoride can be mechanically removed by the action of saliva or mechanical action. Therefore, an addition of acid formulation is recommended. The acidulated sodium fluoride can form precipitates deep inside the tubules. Also, some authors have recommended the use of ionotophoresis along with sodium fluoride.[41,42] The electric current is supposed to increase the ion diffusion. A clinical study has shown that 0.4% stannous fluoride along with 0.717% of fluoride can provide an immediate affect after a 5 minute professional application.[43] Stannous fluoride acts in a similar fashion as that of sodium fluoride, i.e., formation of calcium fluoride precipitates inside tubules. Also, SEM studies have shown that stannous fluoride itself can form insoluble precipitates over the exposed dentine.[44] Fluorosilicates act by formation of precipitates of calcium phosphates from saliva. Ammonium hexafluorosilicate has been used as a desensitizing agent. It can present a continuous effect of dentinal tubule occlusion via precipitation of a mixture of calcium fluoride and fluoridated apatite.[45,46] If the precipitate is predominantly composed of fluoridated apatite, it can form stable crystals deposited deep inside the dentinal tubules.[45,46] These crystals are resistant to removal from the action of saliva, brushing or action of dietary substances.

### Oxalates

Oxalates can reduce dentinal permeability and occlude dentinal tubules. Thirty percent potassium oxalate had shown a 98% reduction in dentinal permeability.[47] Also, topical application of 3% potassium oxalate reduced DH after periodontal therapy.[47] The oxalate reacts with the calcium ions of dentine and forms calcium oxalate crystals inside the dentinal tubules as well as on the dentinal surface. This results in a better sealing as compared with an intact smear layer.[17] It has been shown that the effect of oxalates on DH diminishes over a period of time. This can be attributed to the removal of the calcium oxalate crystals by brushing or dietary acids. The condition can be improved by acid etching of the dentinal surface, thus increasing the penetration of calcium oxalate crystals deep into the dentinal tubules.[48] Many vegetables like rhubarb, spinach and mint contain oxalates. It has been shown that phytocomplexes obtained from these natural products can reduce the dentinal permeability. This can also be followed by covering the exposed surface with a dental adhesive.[49] Potassium oxalate can lead to gastric irritation. Therefore, it should not be used with a tray with prolonged placement.

Varnishes are commonly used useful in-office measures to treat DH. Copal varnish can be applied to cover the exposed dentinal surface. But its effect is for short term and is not recommended for long term management of DH.[50] To improve its efficacy, removal of smear layer is advocated. Also, the varnishes can act as a vehicle for fluoride. The fluoride varnishes can be acidulated to increase the penetration of ions.[50]

### Adhesive materials

Resin-based dental adhesive systems can provide a more durable and long lasting dentine desensitizing effect. The adhesive resins can seal the dentinal tubules effectively by forming a hybrid layer.[17] Various clinical studies have demonstrated the effectiveness of adhesives in management of DH.[51–53] Traditionally, resin composites or dentin bonding agents are used as desensitizing agents. The conventional dentin bonding agents (DBA) removes the smear layer, etches the dentinal surface and forms deep dentinal resin tags inside the dentinal tubules. The combined dentin–resin layer (consisting of penetrating resinous tags) has been termed as hybrid layer. It effectively seals the dentinal tubules and prevents DH.[51–53] Newer bonding agents modify the smear layer and incorporate it in into the hybrid layer.[54] Recently, some dentin bonding agents have been introduced in the market with the sole purpose of treating DH. Gluma Desensitizer (Heraeus Kulzer, Hanau, Germany) contains hydroxyethyl methacrylate (HEMA), benzalkonium chloride, gluteraldehyde and fluoride. Gluteraldehyde causes coagulation of the proteins inside the dentinal tubules.[54] It reacts with the serum albumin in the dentinal fluid, causing its precipitation. HEMA forms deep resinous tags and occludes the dentinal tubules.[54] Gluma has shown promising results in the clinical trials.[54,55]

### Bioglass

Bioglass was developed to stimulate the formation of new bone.[56] It is used in orthopedics to cover the implants to promote union between implant and bone.[56,57] It has been used in dentistry to fill up the osseous defects during periodontal surgery.[58] It has been reported that a formulation of bioglass can promote infiltration and remineralization of dentinal tubules.[59] The basic component is silica, which acts as a nucleation site for precipitation of calcium and phosphate. SEM analysis has shown that bioglass application forms an apatite layer, which occludes the dentinal tubules.[59] The use of bioglass in management of DH has been shown by some products such as NovaMin (NovaMin Technology Inc., FL, USA).

### Portland cement

Some authors have shown that calcium silicate cement derived from Portland cement can help in the management of DH.[17] It helps to occlude the dentinal tubules by remineralization.

### Laser

Laser is an acronym for light amplification by stimulated emission of radiations. It has been shown in various studies that lasers can be used in the effective management of DH.[60–63] The mechanism of action of lasers in treating DH is not very clear. Some authors have shown that Nd–YAG laser application occluded the dentinal tubules.[61,62] GaAlA laser is thought to act by affecting the neural transmission in the dentinal tubules.[63] It has also been proposed that lasers coagulate the proteins inside the dentinal tubules and block the movement of fluid.[61]

### Casein phosphopeptide–amorphous calcium phosphate

Recently, milk protein casein has been used to develop a remineralizing agent (GC Tooth Mousse). The casein phosphopeptide (CPP) contains phosphoseryl sequences which get attached and stabilized with amorphous calcium phosphate (ACP).[64] The stabilized CPP–ACP prevents the dissolution of calcium and phosphate ions and maintains a supersaturated solution of bioavailable calcium and phosphates.[64] Various studies have shown that CPP–ACP can effectively remineralize the enamel subsurface lesions.[65,66] By virtue of its remineralizing capacity, it has also been proposed by the manufacturers that it can also help in prevention and treatment of DH.

## MANAGEMENT STRATEGY

- Take a detailed clinical and dietary history.
- Differentially diagnose the condition from other dental pain conditions.
- Identify and manage etiological and predisposing factors.
- In case of mild-to-moderate sensitivity, advice at-home desensitizing therapy.
- If there is no relief or in case of severe sensitivity, initiate in-office treatment.
- In extreme cases, if patient does not respond to the therapy and there are individual teeth exhibiting the symptoms, then endodontic therapy can be initiated.
- A regular review should be made with an emphasis on prevention of the condition.
